# Antifungal Volatilomes Mediated Defense Mechanism against *Fusarium oxysporum* f. sp. *lycopersici*, the Incitant of Tomato Wilt

**DOI:** 10.3390/molecules27113631

**Published:** 2022-06-06

**Authors:** Praveen Thangaraj, Akshaya Subbaih Balamurali, Krishnamoorthy Akkanna Subbiah, Nakkeeran Sevugapperumal, Thiribhuvanamala Gurudevan, Sivakumar Uthandi, Haripriya Shanmugam

**Affiliations:** 1 Department of Plant Pathology, Tamil Nadu Agricultural University, Coimbatore 641 003, India; akshayaagri14@gmail.com (A.S.B.); nakkeeranayy@tnau.ac.in (N.S.); ragumala2000@gmail.com (T.G.); 2 Department of Agricultural Microbiology, Tamil Nadu Agricultural University, Coimbatore 641 003, India; usivakumartnau@gmail.com; 3 Department of Nanoscience and Technology, Tamil Nadu Agricultural University, Coimbatore 641 003, India; haripriya.s@tnau.ac.in

**Keywords:** volatilomes, phyto-fumigants, antifungal action, *Fusarium oxysporum*

## Abstract

In this study, the volatilomes of naturally growing plant leaves were immobilized in a suitable substrate to enhance vapors’ diffusion in the soil to eradicate the *Fusarium* wilt pathogens in Tomato. Volatilomes produced by *Mentha spicata* leaves immobilized in vermiculite ball was found to be effective and exhibit 92.35 percent inhibition on the mycelial growth of *Fusarium oxysporum* f. sp. *lycopersici* (FOL). Moreover, the volatilomes of *M. spicata* immobilized vermiculite balls were tested based on the distance traveled by the diffused volatilomes from the ball and revealed that the volatilomes of *M. spicata* traveled up to 20 cm distance from the center of PVC (Polyvinly chloride) chamber showed maximum reduction in colony growth of FOL at 12th day after inoculation. Tomato plants inoculated with FOL revealed increased expressions of defense gene, pathogenesis related protein (PR1) with 2.63-fold after 72 h and the gene, transcription factor (WRKY) increased with 2.5-fold after 48 h on exposure to the volatilomes of *M. spicata* vermiculite balls. To the best of our knowledge, this is the first report on development of volatilomes based vermiculite ball formulations. This result indicated that the volatilomes of *M. spicata* are promising phyto-fumigants for management of Tomato *Fusarial* wilt.

## 1. Introduction

Wilt caused by *Fusarium oxysporum* f. sp. *lycopersici* (Sacc.) (FOL) is a devastating disease infecting tomatoes at all stages from seedling to fruiting around the world. FOL being hemi-biotrophic xylem colonizing pathogen, exhibits a wider host range inducing characteristic symptoms such as chlorosis of leaves, browning of vascular tissue and death of plants. In India, a huge loss of up to 45 percent in tomato crops grown under greenhouse conditions was recorded due to FOL incidence [1,2,3]. A considerable yield loss occurs due to the prolonged survival of the pathogen propagules in soil, their rapid germination and infection of plants under conducive conditions.

Cultural practices and regular applications of chemical fungicides are partially effective; however, application of fungicides repeatedly may cause damage to the natural ecosystem, human health and aquatic life, and also be detrimental to the beneficial microflora in soil [4]. Soil disinfestation (SD) by fumigation with highly toxic synthetic chemicals may lead to the development of fungicidal resistance in plant pathogens. However, carbon disulfide, methyl bromide (MB), methyl iodide, formaldehyde and hydrogen cyanide were also banned because of safety concerns and due to their effect on depletion of the ozone layer [5].

Large-scale application of beneficial microorganisms with antagonistic properties against plant pathogens could maintain the ecosystem more productive; keep the crop healthy and develop sustainable crop protection and production [6,7]. Thus, bio-control agents are considered to be the best alternatives for the management of soil-borne fungal pathogens by replacing synthetic chemical fungicides. Although these bio-inoculants enforce several modes of action, the novel concept of plant disease management through Volatile Organic Compounds (VOCs) becomes an attractive topic for research and debate. VOCs produced by the natural plant and microbial origins will have a greater stakeholder in the management of soil-borne plant pathogens. VOCs could have different biological and ecological functions in solemnizing defense mechanisms against plant pathogens and even plant growth promotion [8,9,10].

VOCs are low molecular weight, carbon structured organic compounds with high vapor pressure and lipophilic character [11]. VOCs are chemically diverse and belong to the large group of terpenoids and their derivatives, fatty acid-derived volatiles, phenyl propanoid aromatic compounds, alkanes, alkenes, alcohols, benzenoids, pyrazines, acids, esters, aldehydes, ketones and sulfur-containing compounds [12]. Some VOCs act as signaling mediators for cell-to-cell communications. These compounds are naturally produced through diverse biosynthetic pathways such as methylerythritol phosphate pathway (MEP), fatty acid metabolism, shikimate pathway, mevalonic acid pathway (MVA), lipo-oxygenase pathway (LOX), cinnamate pathway and amino acid oxidation [13,14].

VOCs produced by odorous plants and microbes have received more attention in the management of plant disease, plant growth promotion and induction of defense genes in plants [15]. VOCs extracted from the leaves of *Achyranthes aspera, Lawsonia inermis*, and *Mimosa pudica* [16,17] have been explored for the management of fungal pathogens. VOCs of essential oil such as *Foeniculum vulgare*, *Laurus nobilis*, *Lavandula stoechas* subsp., *stoechas*, *Origanum syriacum* var. *bevanii*, *Rosmarinus officinalis*, and *Thymbra spicata* subsp., *spicata* showed antifungal activities against *Phytopthora infestans* [18]. Similarly, VOCs produced by some microbial biocontrol agents and endophytic fungi act as elicitors that prime defense in plants against pathogens and promote growth [19]. VOCs produced by *Bacillus pumilus* exhibited antimicrobial activities against *F. oxysporum* f. sp. *lycopersici* and also elicited defense mechanisms by up-regulation of defense genes [20]. Despite the efficacy of VOCs in enhancing plant defense under in vitro laboratory, their effectiveness to enhance defenses mechanism against plant diseases has not been explored in the field. To date, a VOCs-mediated push–pull method of management strategy has been developed for attraction and repulsion of herbivores from the plants [21]. Recently, new techniques on micro encapsulation of VOCs have been developed, which allow controlled release of synthetic blends of VOCs, but their efficacy was not known, since it is not yet clear how VOCs inhibit plant diseases in nature.

With this background, the present study was carried out to develop suitable plant and microbes associated volatilome formulations for the management of *Fusarium* wilt pathogen infecting tomato crops under protected cultivation.

## 2. Results

### 2.1. Volatilomes Formulation

In earlier studies, the natural plant and microbe-associated volatilomes were screened individually against *FOL* using a sealed plate assay. The volatilomes produced by the leaves of *Mentha spicata*, *Cymbopogon citratus* and mycelia of *Trichoderma asperellum* showed the maximum higher inhibitory potential on mycelial growth of *FOL.* The leaves of *M. spicata* and *C. citratus* and the mycelial culture of *T. asperellum* were individually immobilized in a vermiculite ball in the form of Volatilomes Immobilized Vermiculite Ball formulation and further tested against the mycelial growth of pathogen under in-vitro. The vermiculite was served as volatile absorbent material.

**Figure 1:**
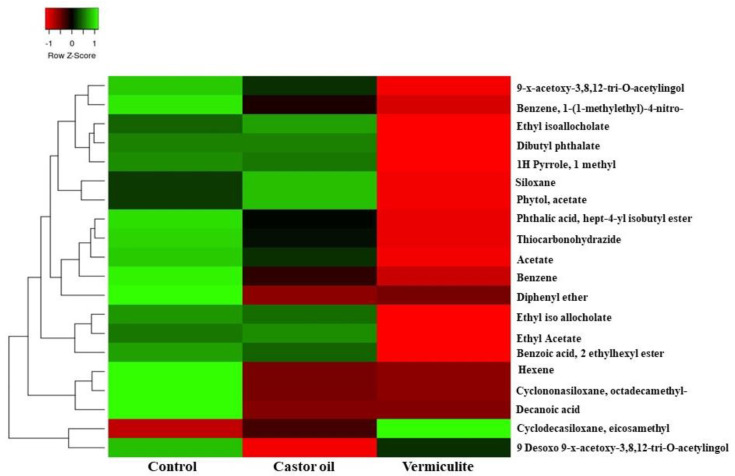
Comparative analysis of VOCs produced by vermiculite and castor oil.

Moreover, the volatilomes produced by vermiculite and castor oil used in the volatilome ball formulation did not show any relative abundance peak area, apart from the column contaminants as displayed in the heatmap (Figure 1). The prepared vermiculite ball-based volatilome formulations were well packed in an airtight zip lock polybag to prevent the diffusion of volatilomes.

### 2.2. Efficacy of Volatilomes Immobilized Vermiculite Balls against Pathogens

**Figure 2:**
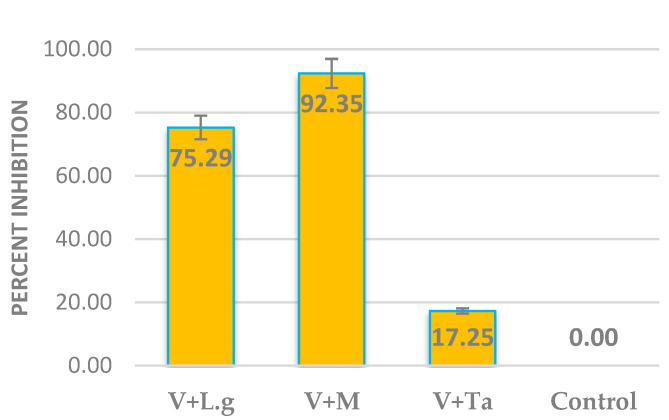
In vitro efficacy of VOCs immobilized vermiculite ball using olfactory chamber, V—Vermiculite, L.g—*C. citratus*, M—*M. spicata*, Ta—*Trichoderma asperellum*.

**Figure 3:**
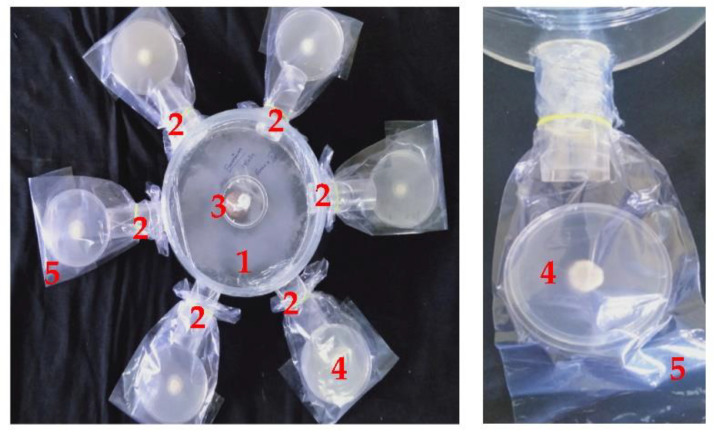
Effect of volatilomes immobilized vermiculite ball using olfactory chamber. 1—Olfactory chamber, 2—Outlet flow of chamber (6 no’s), 3—Vermiculite ball formulation, 4—Petri dish containing pathogen and 5—Sterilized ploy cover.

The efficacy of volatilomes immobilized vermiculite balls were tested against *FOL* using an olfactory chamber. The vermiculite balls of *M. spicata* volatilomes inhibited up to 92.35 percent on the mycelial growth of *FOL* (Figure 2). Vaporous inhibitory action was conspicuous at all six outlets, indicating the diffusion of volatiles all around the olfactory chamber. Subsequently, the vermiculite ball of *C. citratus* leaves showed 75.29 percent inhibition on the mycelial growth of *FOL*. The mycelia of *FOL* were completely altered with distorted growth on experimentation with the olfactory chamber (Figure 3), as compared to normal linear thick, pink-colored mycelial growth in control plates (Figure S1). Conclusively, the mycelial growth and sporulation behavior of the *FOL* was inhibited on exposure to the volatilomes of *M. spicata* leaves immobilized in the vermiculite balls.

### 2.3. GC MS Analysis of M. spicata Volatilomes Immobilized Vermiculite Ball

Based on the significant antagonistic potential on the inhibition of mycelial growth as well as sporulation behavior of *FOL*, the volatilomes immobilized ball of *M. spicata* and *C. citratus* were subjected to GC-MS analysis to find out the presence of major antifungal compounds.

**Figure 4:**
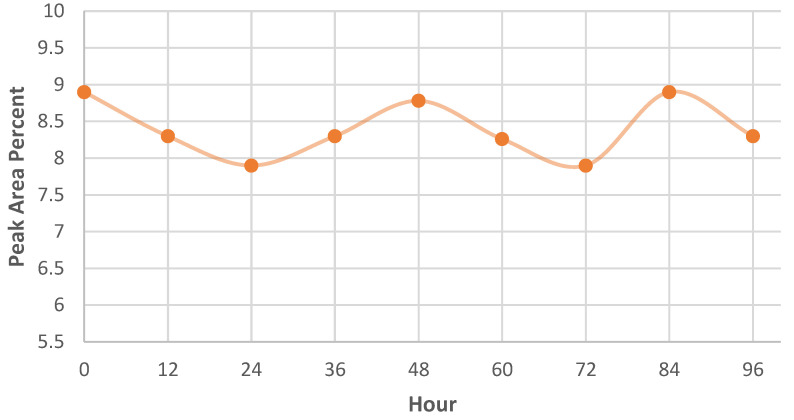
Peak area percent of carvone volatiles produced by the leaves of *M. spicata* immobilized vermiculite ball.

In *M. spicata* immobilized ball, carvone was frequently represented with a peak area of 8–8.26% at 10.23 RT (Figure S2) up to 96 h of trapping (Figure 4, Table S1), which was comparatively less as compared to the peak area of 56% at 9.89 RT when standard carvone was used. The volatilomes of *C. citratus* showed the least diffusion of citronellol volatiles with less peak area of 0.98% at 9.79 RT, which was comparatively very less in comparison to that of the peak area of 31.82% at 9.96 RT, when standard citronellol was used. Therefore, the volatilomes of *M. spicata* ball recorded the maximum diffusion of carvone volatiles with a high peak area percent. However, the volatilomes produced by *C. citratus* (Table S2) did not diffuse citronellol volatiles with similar peak area percent as represented by the standard citronellol. Based on their higher antifungal activity against the pathogen, the volatilomes of *M. spicata* leaves immobilized vermiculite ball was taken to test their efficacy under pot culture conditions.

### 2.4. SEM Imaging of M. spicata Volatilomes Immobilized Vermiculite Ball

**Figure 5:**
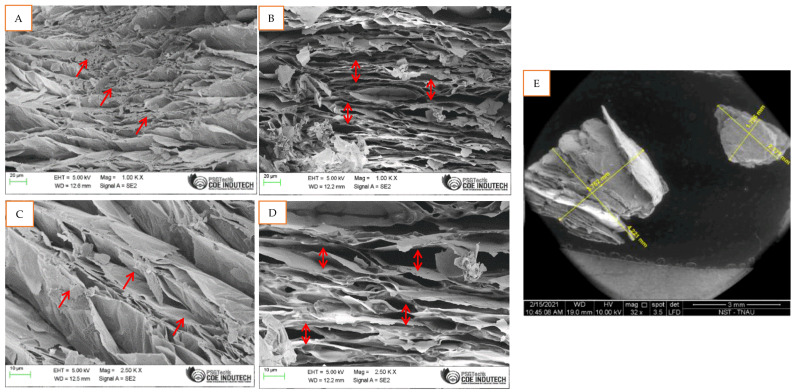
SEM imaging of *M. spicata* vermiculite ball. (**A**,**C**)—Control (Without immobilized) at 1.00 Kx magnification; (**B**,**D**)—*M. spicata* immobilized vermiculite ball at 2.50 Kx magnification. The double arrow indicates expanding, and the single arrow indicates non-expanding. (**E**)—Fold change in control (**Right**) and *M. spicata* (**Left**) immobilized vermiculite ball.

The structural changes of vermiculite loaded with the leaves of *M. spicata* were recorded concerning vertical and horizontal sheet expansion of vermiculite content. The vermiculite sheet ultra-structure was found to be raised by 2.7 folds. The volatilomes immobilized formulation consisting of vermiculite sample for its superior volatile absorbing capacity, to which finely grounded leaves of *M. spicata* was added and blended with castor oil. The volatile permeability and diffusion coefficients of the *M. spicata* immobilized vermiculite balls were altered due to the high loading of vermiculites compared to vermiculite balls without *M. spicata* volatilomes (Figure 5A,C). The volatilomes permeability was increased by 2–3 fold by the vermiculite (Figure 5E). The expansion coefficients of vermiculite were computed from *M. spicata* leaves immobilized ball, and the crystal structure of vermiculite was increased (Figure 5B,D). The expansion of crystal structure from the volatilomes of *M. spicata* ball significantly increased the absorption efficiency with vermiculite content. This expansion is mainly due to a change in the structure of the octahedral and tetrahedral layer, when volatilomes were immobilized in a vermiculite sample in the form of a ball. The excess sorption appears to be the effect of absorption of *M. spicata* volatilomes by the vermiculite and further helps to find out their efficacy against *FOL*.

### 2.5. Testing the Distance Traveled by the Volatilomes Immobilized Vermiculite Ball

In this study, the volatilomes of *M. spicata* immobilized vermiculite ball were kept at the center of the PVC chamber to find out the exact distance traveled by the volatile that diffused from the ball.

**Figure 6:**
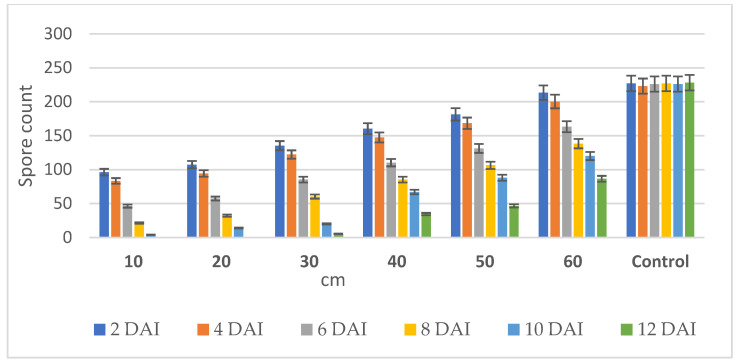
Performance of *M. spicata* volatilomes diffusion from the vermiculite ball over a distance against *F. oxysporum* f. sp. *lycopersici*.

The volatilomes of *M. spicata* balls recorded the maximum inhibition of colony growth of *FOL* at 10 and 20 cm distances away from the center of the PVC chamber. This indicates that *M. spicata* leaves immobilized vermiculite ball traveled in the PVC chamber up to a distance of 20 cm and suppressed the colony growth of the pathogen (0 × 10^−3^ CFU at 12 DAI (Day After Inoculation)) than in control (228 × 10^−3^ CFU) (Figure S3). However, the volatilomes did not travel at a distance above 30 to 60 cm from the center of the PVC chamber and exhibited the highest number of colony regrowth of *FOL* (213 × 10^−3^ CFU on 2 DAI; 87 × 10^−3^ CFU on 12 DAI) (Figure 6). However, the volatilomes of *M. spicata* leaves immobilized vermiculite balls proved to have fungicidal action in reducing the spore count of *FOL* up to a distance of 20 cm from the center of the PVC chamber. From these results, it is inferred that the volatilomes of *M. spicata* balls need to be placed at a distance of 20 cm from the root zone of tomato plants to reduce the infections and pathogen populations present in the soil.

### 2.6. Effect of Volatilomes Immobilized Vermiculite Balls under Glasshouse Condition

In this investigation, Phytoformulation of *M. spicata* volatilomes immobilized vermiculite ball was used to validate their efficacy against *FOL* in tomato under glass conditions using a controlled volatile growth chamber. Among the treatments, *M. spicata* immobilized balls recorded a lesser wilt incidence with 8.33 percent than in control (100 percent wilt incidence) (Table 1). The pathogen-inoculated pot expressed and observed with the symptom of chlorosis, drying of the vascular system followed by wilting of the plants after 45 days of inoculation (Figure S4). The growth parameters were highly promoted on exposure to the volatilomes of *M. spicata* immobilized vermiculite balls with 9.33 no of branches, shoot (31.83 cm) and root length (22.67 cm) as compared to pathogen-un-inoculated and pathogen-inoculated tomato plant.

### 2.7. Defense Genes Expression

The tomato plants on exposure to *M. spicata* leaves immobilized vermiculite balls and challenge inoculated with pathogens (*FOL*) were analyzed for their expression with further quantification of defense genes by using reverse transcriptase quantitative PCR (q-RT PCR). During the quantitative analysis of the PCR product, the melt curve analysis of each gene was performed to eradicate non-specific amplification of the sample. To understand the plant sample-based volatilomes mediated defense mechanisms in tomato plants, four defense responsive genes *viz.*, WRKY transcription factor (WRKY), thaumatin-like protein (TLP), pathogenesis-related protein (PR1) and lipoxygenase (LOX) were studied for their expression patterns. All the genes that were focused on were found to be up-regulated in tomato plants after 48 and 72 h on exposure to *M. spicata* volatilomes immobilized vermiculite balls. Plants challenge inoculated with pathogens and those which are exposed to volatilomes of *M. spicata* recorded higher levels of expression patterns of all the four defense responsive genes studied.

**Figure 7:**
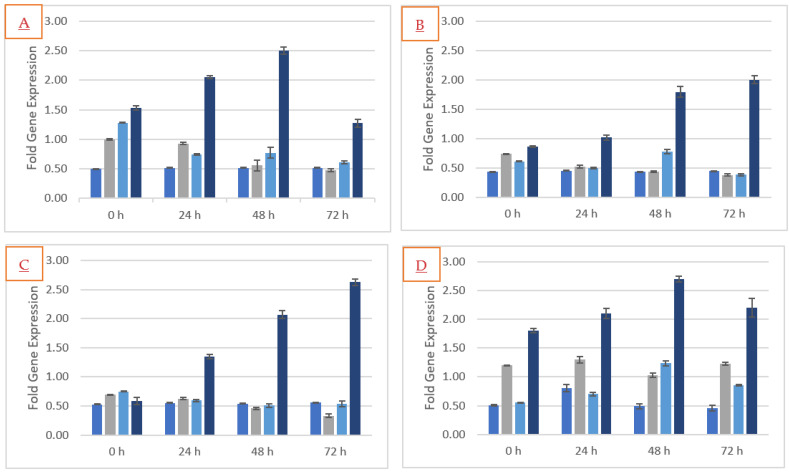
Defense gene expression of (**A**) WRKY, (**B**) TLP, (**C**) PR1 and (**D**) LOX during interaction of volatilomes of *M. spicata* immobilized vermiculite ball with *F. oxysporum* f. sp. *lycopercisi* inoculated Tomato Plant.

Tomato seedlings exposed to the volatilomes of *M. spicata* immobilized vermiculite ball formulation and challenges inoculated with *FOL* were assessed for the expression of *SlWRKY*, *SlTLP*, *SlPR 1* and *SlLOX* gene in roots due to the tripartite interactions. The relative quantification of the defense gene was normalized to *SlACTIN*. Among the different HPT, the volatilomes of *M. spicata* exposed tomato plants and challenge inoculated with pathogen highly induced the expression of *SlWRKY* gene at 48 HPT (2.5-fold) followed by pathogen-un-inoculated with plants (0.67-fold). The pathogen-un-inoculated and inoculated tomato plants showed a lower level of *SlWRKY* gene expression during all hours of post treatment. However, during the tri-trophic interaction, i.e., volatilomes of *M. spicata* exposed plants infected with the pathogen showed an up-regulation of the expression of the *SlWRKY* gene (Figure 7A), which conclusively indicated that the volatilomes of *M. spicata* could trigger and activate defense mechanism in tomato plants. The defense gene *SlTLP* was expressed at 72 HPT (2.00 fold) and 48 HPT (1.80 fold). A lower level of *SlTLP* gene expression was displayed in all the treatments at 0 and 24 HPT. This result indicated that the volatilomes of *M. spicata* up-regulated the expression of *SlTLP* gene in pathogen-inoculated tomato plants (Figure 7B). In the case of the defense gene *SlPR1*, higher level of *SlPR1* expression at 72 HPT (Figure 7C) was followed by 48 HPT (2.63-fold and 2-fold, respectively). Hence, the volatilome of *M. spicata* exposed plants significantly up-regulated the *Sl*PR1 gene, showing the potential of priming defense against pathogen infection. Similarly, the higher level of expressions of *SlLOX* gene was observed at 0, 24, 48 and 72 HPT on the volatilomes of *M. spicata* exposed plant. Meanwhile, the pathogen-un-inoculated, pathogen-inoculated and the volatilomes of *M. spicata* exposed un-inoculated plants showed a lower level of *SlLOX* gene expression (Figure 7D). Nevertheless, the volatilomes of *M. spicata* exposed plants strongly expressed the activation of immune response in tomato plants. Conclusively, the results indicated that the tomato plants exposed to volatilomes of *M. spicata* triggered a resistance mechanism against *F. oxysporum* f. sp. *lycopersici*. 

## 3. Discussion

As reported by several authors, the natural plant and microbial samples are well known to produce a wide variety of antimicrobial activity, as they are naturally active against a large spectrum of phytopathogenic microorganisms [22,23]. In the present study, the antifungal volatile of *M. spicata*, *C. citratus* and mycelia of *T. asperellum* was found to be effective on the mycelial growth of *FOL.* Based on this significant inhibition of the pathogen, the volatilomes produced by the leaves of *M.* spicata and *C. citratus* and mycelial cultures of *T. asperellum* were immobilized in a vermiculite ball-based formulation for further testing under pot culture conditions.

Suitable immobilization substrates have been developed to induce high vaporous diffusion of volatile compounds. [24] reported that vermiculite coated copper nanoparticle showed the maximum antifungal activity against the pathogens. Earlier studies reported that vermiculite could act as a superabsorbent composite and it swelled rapidly on increasing the concentration of volatilomes [25]. Vermiculite based nanoparticles, coated with CuO and ZnO could act as antibacterial activities against *Staphylococcus aureus* with long-acting up to 24 h [26,27]. In the existing study, vermiculite was used as a volatilomes immobilizing substrates in the form of organic-based formulation against the fungal pathogens. The SEM study also proved that the vermiculite sheet was expanded two fold when immobilized with *M. spicata* leaves compared to the vermiculite ball without volatilomes.

Furthermore, the volatilomes of *M. spicata* leaves immobilized vermiculite balls effectively inhibited the mycelial growth of *FOL* (92.35 percent) using an olfactory chamber. Leaves of *C. citratus* immobilized vermiculite balls showed 75 percent inhibition of *FOL*. However, this was the first report on mycelial growth inhibition of the pathogens using an olfactory chamber assay. In contrast to the present investigation, an olfactory chamber mediated assay was reported to attract and repel the insect population using novel volatile compounds [28]. VOCs, 1-octen-3-ol and benzaldehyde were produced by lemongrass oil, which attracts and repels *Aedes aegypti* mosquitos [29]. Additionally, the volatilomes of *M. spicata* and *C. citratus* were quantified based on the production of the highest area percent of major VOCs. In support to the present findings, [30] also recorded that VOCs of carvone was highly produced by diverse plant samples, but greater in *M. spicata,* and also validated that less peak area percent of citronellol was produced by *C. citratus* than earlier reported *C. nardus* [31]. Hence, our study proved that the vermiculite balls immobilized with *M. spicata* produced carvone volatiles with constant production of relative peak area abundance (8.00%) even up to 96 h, which could effectively suppress *FOL* in tomato plants.

In addition, the defense responsive gene used in the present study could explore a significant role in the activation of defense mechanisms in tomato plants. Gene responsible for the induction of salicylic acid (SAR) and pathogenesis-related protein (PR 1) are widely used as molecular markers and greatly up-regulated when exposed to VOCs as reported by [32]. It has been reported that volatiles of 3-pentanol exposure strongly induced the up-regulation of the pathogenesis-related protein PR 1, PR 2 and proteinase inhibitor PIN 2 during the pathogen attack in the pepper plants [33]. In the present study, *M. spicata* immobilized vermiculite balls when exposed to pathogen-inoculated tomato plants exhibited the highest level of LOX gene expressions. The expression of *Sl*LOX gene steadily increased after inoculation with *FOL* (2.7-fold). It has been reported that the expression of LOX gene led to the activation of the jasmonic acid pathway on interaction with volatilomes of *M. spicata*. The higher RNA transcript level was observed at 48 HPI and increases after 72 HPI in tomato plants. The defense gene expression pattern of *Sl*LOX gene varied significantly between the treatments as reported by [32]. The authors of [34] reported that broad beans inoculated with *Puccinia striiformis* f. sp. *tritici* induced the expression of PR 1 genes. This was evident from the present findings that up-regulation of *Sl*PR1 genes was increased in tomato on interaction with volatilomes of *M. spicata* in the presence of pathogens. This result supports the hypothesis that the induction of the SAR pathway may be responsible for the activation of the resistance mechanism in tomato plants to pathogen attacks. In tomato, defense expression gene LOX and PR-1 were up-regulated after exposure with volatilomes of *M. spicata* and challenge inoculated with pathogen during 48 and 72 HPT, as observed by [32]. Thus, VOC-treated plants against *FOL* expressed a higher level of PR-1 gene through an SA-dependent signaling pathway. The present findings are further supported by [35], who reported that VOCs produced by *Ampelomyces* and *Cladosporium* prime the defense mechanism by activating the salicylic acid and jasmonic acid pathway in *Arabidopsis*. The volatiles of methyl benzoate (MeBA) and m-cresol treated in the *Arabidopsis* plant activates the defense-related genes PR1. Similarly, VOCs of methyl benzoate produced by *T. asperellum* induced the expression of the PR1 gene in *Arabidopsis* plants [36]. As for *Sl*PR 1 gene response, the defense responsive gene *Sl*WRKY was expressed in the present study on treatment with the volatilomes of *M. spicata* after 48 and 72 HPT in pathogen-inoculated tomato plants. This indicates the activation of the defense gene (*Sl*WRKY) in plants against pathogens as observed by [32]. The present findings hypothesize that expression of the *Sl*WRKY gene in tomato inoculated with *FOL* might induce the activation of the gene containing W box in the promotors as reported by [37]. Thaumatin-like proteins (TLP) are a highly complex protein family, which plays a significant role in amino acid composition and induces defense mechanisms in plants. The expressions of the TLP gene could strongly reduce pathogen infections in plants [38]. In the current study, the defense gene *Sl*TLP differentially expressed after 24 HPT and considerably increased on 48 HPT on interaction with volatilomes of *M. spicata* and it might be acted as an elicitor. Conclusively, the defense-related gene such as *Sl* WRKY, *Sl* PR 1, *Sl* LOX, and *Sl* TLP were differentially expressed on interaction with vermiculite ball of *M. spicata* with tomato plants after 48 HPT. Among the treatments, a low level of defense gene expression was noticed in volatilomes exposed tomato plants without pathogens. 

Hence, the volatilomes of *M. spicata* vermiculite balls induced all the defense genes in tomato plants, but the maximum level of defense gene expressions was more in *FOL* inoculated plants than un-inoculated tomato plants. It might be attributed due to the activation of systemic acquired resistance in the tomato plants when exposed to the volatilomes of *M. spicata* leaves immobilized vermiculite balls.

## 4. Materials and Methods

### 4.1. Plant and Microbial Volatilomes

The volatilomes produced by the leaves of *Mentha spicata, Cymbopogon citratus*, *Vitex negundo,* *Coleus amboinicus*, *Vetiveria zizanioides*, *Ocimum tenuiflorum*, *Azadirachta indica*, mycelia of *Auricularia auriculata*, *Coprinus cinereus*, *Ganoderma lucidum*, *Lentinus edodus*, *Trichoderma asperellum* and cell cultures of *Bacillus subtilis*, *Streptomyces rochei* have been collected, selected and bio-efficacy explored [39]. Among the plant and microbial samples, *M. spicata*, *C. citratus* and *T. asperellum* were taken further to develop a suitable formulation against the mycelial growth of *F. oxysporum* f. sp. *lycopersici*.

### 4.2. Immobilization of Eluted and Trapped Volatile Compounds

Volatile absorbing materials such as expanded vermiculite were used in this study. As a major portion of the void volume of vermiculite, could absorb natural volatiles with high retention properties at optimum temperature, it was further used to develop a novel formulation in the form of balls for the immobilization of eluted and trapped volatilomes. Seven parts of Vermiculite were mixed well with three parts of finely grounded volatile producing leaf sample (7:3 ratios). The well-mixed sample was made into a ball (3 mm diameter) by using one mL of castor oil. The vermiculite and castor oil-associated volatiles were also estimated by following the procedure of volatile trapping as mentioned by [39] and optimized for positive release of volatilomes produced by the plant samples used. The vermiculite based volatilomes immobilized ball formulation was further used to study the interactions with the test pathogens. Further, the volatilomes produced by the leaves of plant samples were trapped and analyzed using air-entrainment technique [39] and immediately subjected to HS-GCMS analysis using Thermo GC injector coupled with a Mass Spectrophotometer. The important VOCs, carvone produced were noticed based on the percent area and probability of compounds at different hours to validate on hours of carvone production. Further, the leaves of *M. spicata* immobilized vermiculite ball were examined under Scanning Electron Microscope (Sigma with gemini column, Carl zeiss (Oberkochen, Germany); Resolution 1.5 nm) to observe the structural changes during volatilomes absorption and diffusion.

### 4.3. Testing the Volatilomes Immobilized Vermiculite Balls against Pathogens

**Figure 8:**
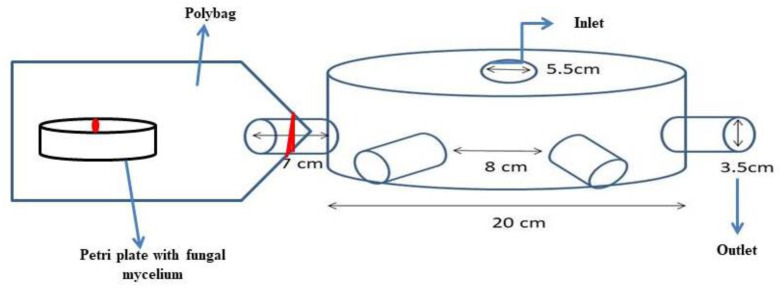
Model of olfactory chamber.

The vermiculite balls immobilized with VOCs were tested for their inhibitory effect against test pathogens by the olfactory chamber method. The olfactory chamber was designed with six outlets on all sides, each measuring 3.5 cm in diameter and an inlet at the center of the chamber with 5.5 cm diameter as shown in Figure 8. Each of the outlets was interconnected to the 20 cm diameter circular chamber, so as to facilitate the free flow of volatiles from the central inlet to all the six outlets. The volatilomes immobilized vermiculite ball was placed at the center of the circular chamber. A 5 mm mycelial disc from 7 days culture of the test pathogens separately, was placed on sterile plastic Petri plates poured with PDA medium. Each of the Petri plates after removing the top lid was placed inside a sterilized poly bag and the mouth of the bag was kept intact with the outlet mouth and sealed airtight. The entire setup with Petri plates was incubated at 28 ± 2 °C until the mycelia in control covered the plate fully. The percent reduction in mycelial growth was recorded by using the formula:

$$\mathrm{Percent}\;\mathrm{inhibition}\;(\mathrm{PI})=\frac{C\;–T\;\;}{C}\times 100$$

where, *C* is the mycelial coverage of pathogen in control, *T* is the mycelial growth of the pathogen in treatment. The experiment was performed thrice with three replications to confirm the efficacy of volatilomes produced by vermiculite ball.

### 4.4. Testing the Distance on Volatilomes Travel

**Figure 9:**
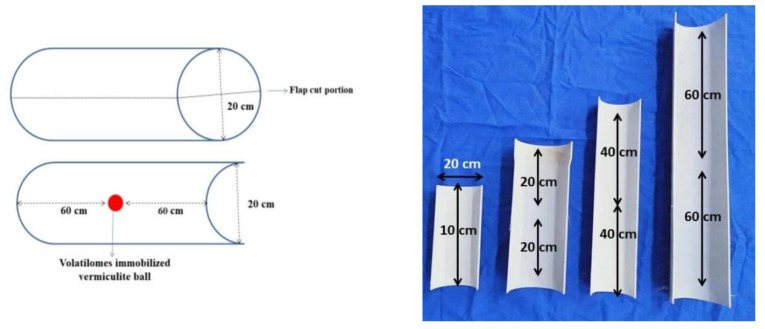
Model of PVC Pipe Chamber at different lengths.

The distance traveled by the volatilomes mixture immobilized in vermiculite balls used against the test pathogens and the efficacy was tested in a PVC chamber. A PVC pipe having 20 cm diameter was cut into different lengths *viz.*, 10, 20, 40 and 60 cm (Figure 9). The PVC pipes were given with a flap cut to 10 cm width and length vary depending upon the size of PVC pipe used. In the flap cut PVC pipe, autoclaved coir compost was filled and inoculated with the spore suspension of the pathogens, separately and incubated for 7 days to induce the mycelial growth of pathogens. Later, one of the vermiculite balls with immobilized VOCs was buried in the pathogen-inoculated coir compost taken in the PVC pipes. The flap-cut portion of the PVC pipe was carefully covered with Parafilm. The experimental setup was incubated at 28 ± 2 °C and replicated thrice for each of the vermiculite ball-based formulations. A similar experimental setup was followed for control by placing vermiculite balls without the volatilomes fraction. After 4 days of incubation, the compost was collected from the PVC pipe at different distances surrounding the vermiculite ball, serially diluted with sterilized water and 10^−3^ dilution was plated on a PDA medium. The CFU count was recorded in both treatment and control at varied distances per interval and tabulated. Based on the results, a separate experiment was conducted on tomato crops grown under the poly house.

### 4.5. Effect of Volatilomes Immobilized Vermiculite Balls Tested in Growth Chamber

**Figure 10:**
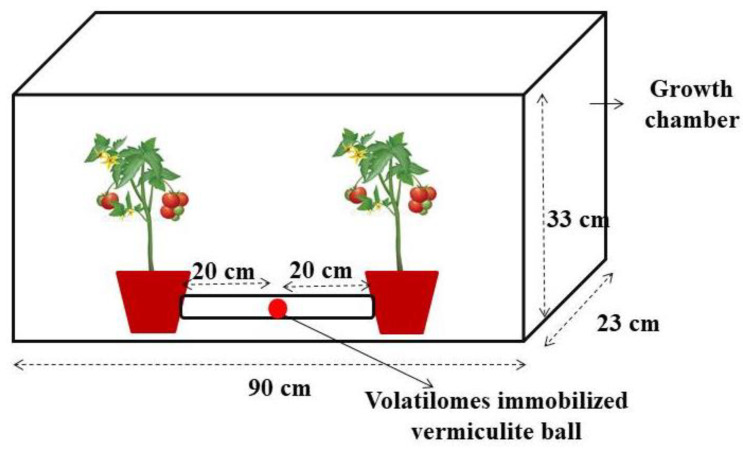
Volatilomes diffusing growth chamber model.

The interaction of volatilomes immobilized vermiculite ball was evaluated under a pot culture experiment in tomato plants. The tomato seedling was transplanted in a pot filled with sterilized coir compost. The pots planted were interconnected with transparent polythene tubes and placed inside the volatilomes diffusing chamber (Figure 10). Arrangements were made prior to placing the volatilomes immobilized vermiculite ball inside the transparent polythene tube facilitating the free flow of volatilomes emitted by the ball between the interconnected pots. The *M. spicata* leaves immobilized vermiculite ball were imposed with four replications. Control pots interconnected with polythene tube but without VOCs immobilized vermiculite ball was maintained inside the chamber as control treatments. The pathogen was inoculated to 20 days old tomato seedlings in treatment (*M. spicata* exposing chamber) and control pots. The pathogen was not inoculated in the Pathogen un-inoculation pot, but the volatilomes of *M. spicata* ball were exposed to the pathogen-un-inoculated pot to compare the difference in volatilomes treated tomato seedling inoculated with the pathogen. Then, the volatile chamber was closed completely until the symptoms are noticed in control pots inside the chamber. The spore suspension of *FOL* was inoculated in a sterilized coir compost and the volatilomes formulation was exposed after a day of pathogen inoculation. The result of percent disease incidence and percent reduction over control was calculated after symptom expression in the pathogen-inoculated pots with treatment. In addition, the growth parameters such as root length, shoot length and number of branches produced by tomato plants were observed.

### 4.6. Defense Gene Expression Studies

A pot culture experiment was conducted with tomato plants (PKM 1) to evaluate the defense genes expression as triggered by the volatilomes immobilized in vermiculite balls containing *M. spicata* leaf volatilomes. The root samples from the plants inoculated with pathogen were taken for determining defense genes expression due to the tripartite interaction between plant, pathogen and immobilized volatilomes. The control plants were maintained with and without pathogen inoculation.

#### 4.6.1. RNA Extraction, cDNA Conversion

Total RNA was extracted separately, from tomato plants under different treatments by following the procedures described by [32]. The root samples were collected on the first day after pathogen inoculation, rinsed in sterile distilled water and dried on filter paper. The dried root samples were weighed (200 g) and ground to a fine powder in a sterilized pestle and mortar using liquid nitrogen. The ground sample was transferred to a microfuge tube (1.5 mL) and added with one mL of cell lysis solution, followed by gentle shaking and centrifugation at 11,000 rpm at 4 °C for 15 min. After centrifugation, the supernatant was transferred to a new microfuge tube and added with 250 µL of phase separation solution. The contents were vortexed well and centrifuged again at 11,000 rpm at 4 °C for 15 min. The top aqueous portion was pipetted out to a new microfuge tube and added with an equal volume of precipitation solution and sodium hydroxide before incubating at −20 °C for 45 min. The precipitated RNA was centrifuged at 11,000 rpm at 4 °C for 15 min and the supernatant was discarded. The pellet was washed by adding one mL of 70 percent ethanol and centrifuged at 11,000 rpm at 4 °C for 15 min. The supernatant was decanted and the pellet air-dried for 10 min and resuspended with 30 µL of sterile distilled water. The quantity and quality of RNA were confirmed using Nanodrop™ 2000 spectrophotometer (Thermo-Fisher, Waltham, MA, USA). The extracted RNA was stored at −80 °C for further studies. 

The DNA admixed in the extracted RNA sample was removed by following the procedure given in DNAse I removal Kit (Sigma Aldrich, Saint Louis, MO, USA). The extracted RNA (8 µL) was treated with one µL each of 10× DNAse reaction buffer and DNAse I. The DNAse treated RNA sample was incubated at 28 ± 2 °C for 15 min. After incubation, the DNAse reaction was stopped by adding 1 µL of EDTA as a stop solution to inactivate the DNAse I and incubated at 70 °C for 10 min. Then, the DNA-free RNA sample was used for cDNA synthesis. A quantity of 12 µL of DNA-free RNA sample was taken for activation of cDNA synthesis. The RNA sample was added with one µL of random primer and incubated at 65 °C for one min in a PCR thermocycler. The incubated contents were placed in ice and 4 µL of 5× reverse transcriptase (RT) reaction buffer; 2 µL of 10 mM dNTP mixture; 1 µL of revert aid were added and the contents were completely mixed and incubated in PCR thermocycler at three different step process: 42 °C for 60 min; 50 °C for 15 min; 70 °C for 15 min. The cDNA synthesized samples were stored at −80 °C.

#### 4.6.2. Quantification of Defense Related Genes Expression

The defense gene expression in tomato plants, both in pathogen-infected and treatment imposed with volatilomes was quantified by quantitative PCR (Bio-rad, California, United States of America) using SYBR Green (KAPA SYBR FAST LC 480). The housekeeping gene ACTIN was used as internal control and the diluted cDNA samples were used for qPCR analysis. Each of the 10 µL reaction mix contained 5 µL SYBR green master mix (KAPA SYBR FAST LC 480, Sigma Aldrich, Saint Louis, MO, USA); 2 µL of primers (Forward and Reverse defense primers); one µL of sterile distilled water and 2 µL of diluted cDNA. The quantification of defense genes was performed in Biorad RT PCR with the following settings: initial denaturation (95 °C for 10 min), 39 cycles of PCR (includes denaturation 95 °C for 30 s and amplification at 58 °C for 30 s), melting curve (includes denaturation 95 °C for 5 s, amplification at 65 °C for 5 s). The relative expression of defense genes was compared with internal control (ACTIN gene) and further assessed. The changes in expression levels were analyzed using the relative quantification (^ΔΔ^Ct) method. The analysis was carried out with three biological replicates and three technical replicates for each of the defense primers. The relative expression of each defense gene was compared among the treatments. The details of defense primers of tomato plants are listed in Table 2.

### 4.7. Statistical Analysis

In-vitro experiments were performed in triplicate and analyzed using a single ANOVA with the data obtained from the radial growth of pathogenic inhibition. The result was presented as mean ± standard deviation. The GC-MS data were statistically analyzed using MetaboAnalyst V5.0 (Genome Canada, Ottawa, ON, Canada). The results of the GC-MS data were presented as a heat map using heatmapper.ca.in.

## 5. Conclusions

In conclusion, volatilomes produced by the leaves of *M. spicata* could induce diverse antifungal VOCs for the management of plant pathogens. Diverse volatile biomolecules have been characterized from the leaves of *M. spicata*, but some VOCs such as carvone play a major role in the suppression of pathogenic microorganisms. VOC of carvone has been proved as antimicrobial activity for the management of plant disease in horticultural crops. Thus, volatilomes produced by *M. spicata* need to be explored.

In the present study, we assessed the various immobilization substrate for volatilomes based product development to eradicate the presence of pathogenic propagules present in the soil. Further, we conclude that volatilomes of *M. spicata* leaves immobilized in a vermiculite ball were found to be effective in managing the plant disease under glasshouse conditions. The present findings could be useful for the precise analysis of *M. spicata* leaves immobilized vermiculite ball against soil-borne plant pathogens.

## Figures and Tables

**Table 1:** Efficacy of VOCs immobilized vermiculite ball against *F. oxysporum* f. sp. *lycopersici*.

Treatments	Shoot Length(cm)	Root Length (cm)	No of Branches	Percent Wilt Incidence	Percent Reduction over Control
Vermiculite + *M. spicata* in pathogen inoculated	31.83	22.67	9.33	8.33 ^c^ (16.78)	91.67 ^a^ (73.22)
Uninoculated control	25.83	15.87	7.33	83.33 ^b^ (65.91)	16.67 ^b^ (24.09)
Inoculated control	21.67	3.10	3.33	100.00 ^a^ (90.00)	0.00 ^c^ (0.02)

Values are the mean of five replications. Data in parentheses are arc sine transformed values. Means in a column followed by the same superscript letters are not significantly different according to the DMRT test at *p* = 0.05. Superscript letters in Percent Wilt Incidence a, b—Higher Wilt Incidence, c—Lower Wilt Incidence. Superscript letters (a, b, c) in Percent Reduction over Control represent higher to lower inhibition of pathogenic propagules in the soil.

**Table 2:** Defense gene primers used for quantitative RT-PCR.

Defense Gene	Forward and Reverse Primer Sequence	Reference
*Sl*PR 1	Forward: 5′-ACGTCTTGGTTGTGCTAGGG-3′	[32]
	Reverse: 5′-TCAAAAGCCGGTTGATTTTC-3′	
*Sl*LOX	Forward: 5′-TGGGATTAAACTGCCAGACC-3′	
	Reverse: 5′-GGCATCGGAAATTTGAGAAA-3′	
*Sl*WRKY	Forward:5′-TCTCGATCTGACCAGGTTCC-3′	
	Reverse: 5′-TTGCCGTCTTCGTTCTCTTT-3′	
*Sl*TLP	Forward:5′-CCATCTTTGCTTCCCACATT-3′	
	Reverse: 5′-ATCGGTTTACCTGCACTTGG-3′	
*Sl*Actin	Forward:5′-AGGCACACAGGTGTTATGGT-3′	
	Reverse: 5′-AGCAACTCGAAGCTCATTGT-3′	

## Data Availability

All the data is available in the manuscript.

## Supplementary Materials

The following supporting information can be downloaded at: https://www.mdpi.com/article/10.3390/molecules27113631/s1, Figure S1: Morphological Characterization of mycelial growth of *Fusarium* Pathogen using olfactory chamber; Figure S2: GCMS chromatogram of headspace volatile compounds produced by the leaves of *Mentha spicata*; Figure S3: Colony growth of *Fusarium oxysporum* (CFU) using PVC chamber; Figure S4: Efficacy of Volatilomes Immobilized Vermiculite Balls under Glass house Condition in Tomato Plant; Table S1: Area Percent of carvone produced at different hours by the leaves of *M. spicata* immobilized vermiculite ball; Table S2: GC-MS profiling of VOCs produced by leaves of *Cymbopogon citratus* immobilized vermiculite ball.
