# Supplementary material for: Antifungal Volatilomes Mediated Defense Mechanism against Fusarium oxysporum f. sp. lycopersici, the Incitant of Tomato Wilt
Source: Molecules. 2022 Jun 6;27(11):3631. doi: 10.3390/molecules27113631 (PMC9182059; doi:10.3390/molecules27113631)
Supplement: Supplementary file 1 [file molecules-27-03631-s001.zip › molecules-1699848-supplementary.pdf]

Supplementary data

Figure S1. Morphological Characterization of Pathogen using olfactory chamber.

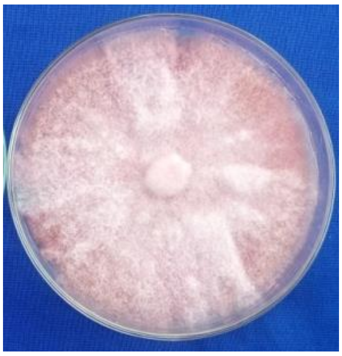

Control (*Fusarium*)

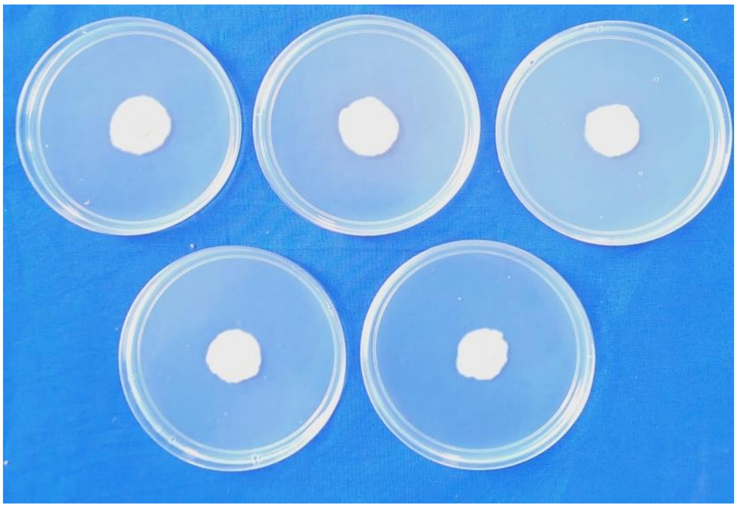

M. spicata volatilomes exposed plates

Figure S2: GCMS chromatogram of headspace volatile compounds produced by the leaves of *Mentha spicata*

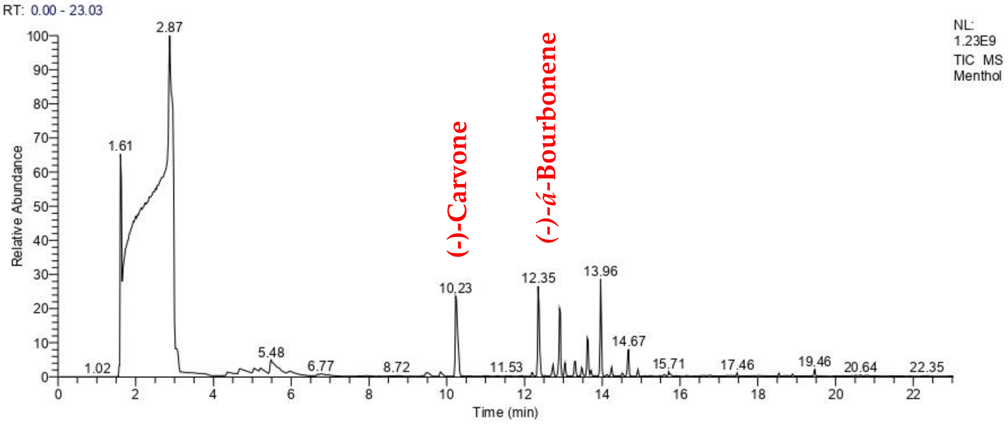

Figure S3. Colony growth of *Fusarium oxysporum* (cfu) using PVC chamber

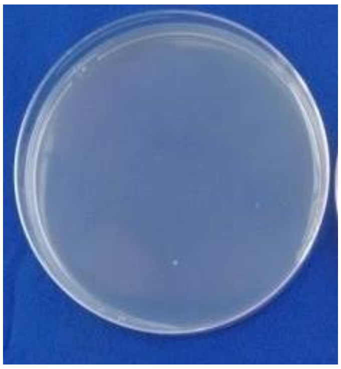

3rd dilution after exposure to 12 days of volatilomes

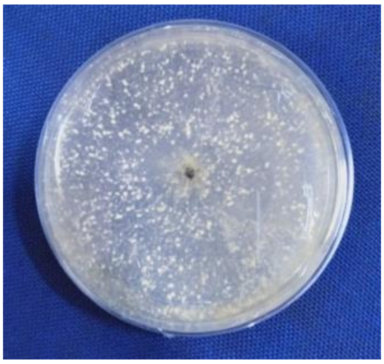

12th day without volatilomes exposure

Figure S4. Effect of Volatilomes Immobilized Vermiculite Balls under Glass house Condition

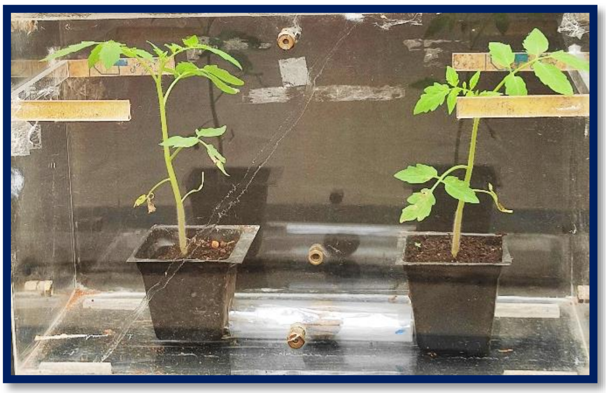

a. *M. spicata* vermiculite ball exposed in pathogen inoculated pot

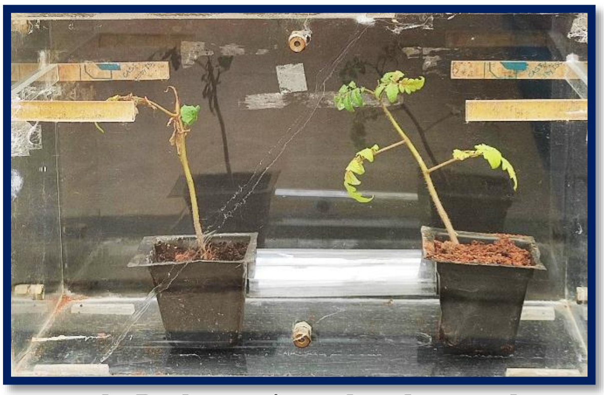

b. Pathogen inoculated control

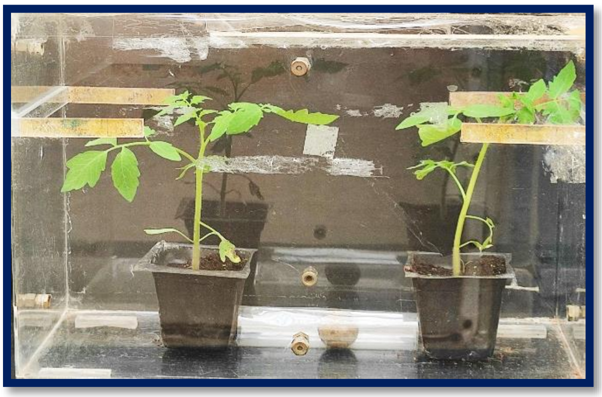

c. Control without pathogen

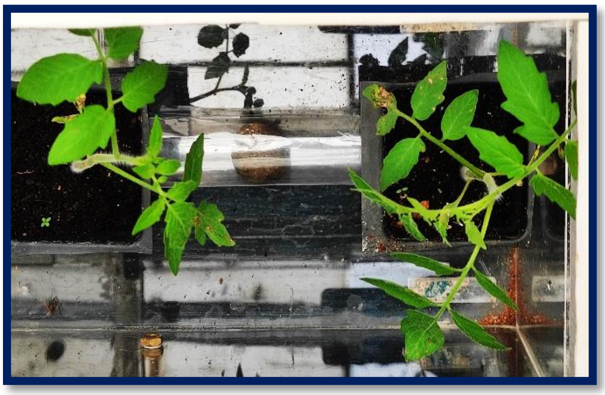

d. Aerial view of Volatile Chamber

Table S1. Area Percent of carvone produced at different hours by the leaves of *M. spicata* immobilized vermiculite ball

Compound	Hours								
	0	12	24	36	48	60	72	84	96
Dodecane	0.069	0.02	0.021	0.31	0.028	0.26	0.021	0.078	0.36
2-Propanamine, N-(phenylmethylene)-	0.613	0.52	0.621	0.601	0.503	0.629	0.267	0.153	0.01
**Carvone**	**8.9**	**8.3**	**8.9**	**8.30**	**8.78**	**8.23**	**8.9**	**8.9**	**8.26**
1-Pentanone,1-(1H-imidazol-4-yl)-	0.141	0.09	0.46	0.068	0.23	0.61	0.51	0.13	0.68
1-Cyclohexene-1-carboxaldehyde, 2,6,6-trimethyl-	0.327	0.281	0.312	0.298	0.356	0.328	0.308	0.37	0.08
(-)-Bourbonene	3.50	2.20	3.431	3.981	1.563	1.031	0.671	0.60	0.31
p-Mentha-1,8-dien-7-ol	0.283	0.286	0.368	0.407	0.48	0.512	0.238	0.73	0.17
Tetradecane	0.278	0.163	0.261	0.387	0.08	0.32	0.810	0.28	0.89
3-hydroxy-2-methyl-5-cyclohexanone	0.192	0.17	0.213	0.298	0.198	0.094	0.230	0.02	0.29
Bicyclo[3.2.1]octan-6-ol, exo-	0.32	0.298	0.13	0.312	0.568	0.138	0.18	0.49	0.02
Disulfide, di-tert-dodecyl	0.116	0.094	0.004	0.098	0.319	0.275	0.063	0.11	0.08
Heptacosane	0.259	0.237	0.78	0.624	0.0642	0.294	0.128	0.09	0.84
Dodecane, 2,6,11-trimethyl-	0.2	0.178	0.128	0.342	0.267	0.162	0.93	0.28	0.31
Hexadecane	0.223	0.201	0.287	0.236	0.128	0.045	0.377	0.83	0.46
Heptacosane	0.219	0.197	0.138	0.102	0.284	0.198	0.012	0.79	0.02
Octadecane	0.98	0.958	0.871	0.231	0.328	0.249	0.73	2.13	0.231
Eicosane, 2-methyl-	0.155	0.133	0.05	0.094	0.194	0.00	0.059	0.17	0.04
Dibutyl phthalate	0.86	0.838	0.793	0.846	0.762	0.83	0.430	0.68	0.80
Eicosane	0.115	0.093	0.16	0.267	0.197	0.23	0.20	0.065	0.427
2-methyloctacosane	0.178	0.156	0.23	0.138	0.08	0.198	0.910	0.108	0.738
Octadecane, 2-methyl-	0.17	0.148	0.05	0.32	0.14	0.128	0.203	0.312	0.701
Tetratetracontane	0.34	0.318	0.36	0.216	0.287	0.321	0.72	0.04	0.25

Table S2. GC-MS profiling of VOCs produced by leaves of *Cymbopogon citratus*

RT	Compound	Molecular formula	Molecular weight	Relative area abundance
2.89	Oxirane, 2-ethyl-2-methyl-	$C_5H_{10}O$	86	0.87
4.75	2,2,4-Trimethyl-3-pentanol	$C_8H_{17}N$	127	0.38
5.12	Hydroperoxide, 1-ethylbutyl	$C_6H_{14}O_2$	118	0.34
5.54	Oxirane, butyl-	$C_6H_{12}O$	100	0.72
7.03	3-Carene	$C_{10}H_{16}$	136	0.05
7.95	1,6-Octadien-3-ol, 3,7-dimethyl-	$C_{10}H_{18}O$	154	0.07
8.78	Isopulegol	$C_{10}H_{18}O$	154	0.16
9.97	**Citronellol**	$\mathbf{C_{10}H_{20}O}$	**156**	**3.27**
10.37	Geraniol	$C_{10}H_{18}O$	154	2.01
11.02	Geranyl vinyl ether	$C_{12}H_{20}O$	180	0.00
12.13	2,6-Octadien-1-ol, 3,7-dimethyl-, acetate	$C_{10}H_{18}O$	154	1.20
12.9	Caryophyllene	$C_{15}H_{24}$	204	0.13
14.55	ç-Muurolene	$C_{15}H_{24}$	204	0.09
15.32	à-acorenol	$C_{15}H_{26}O$	222	0.00
15.59	4-epi-cubedol	$C_{22}H_{32}O_2$	328	0.63
16.22	Cubedol	$C_{23}H_{22}O_6$	394	0.03
16.79	à-Cadinol	$C_{15}H_{26}O$	222	0.13
17.46	Benzoic acid, 2-ethylhexyl ester	$C_{15}H_{22}O_2$	234	0.18
17.97	Geranyl isovalerate	$C_{15}H_{26}O_2$	238	0.04
18.54	Octadecane	$C_{18}H_{38}$	254	0.04
18.54	Heptacosane	$C_{20}H_{60}O_{10}Si_{10}$	740	0.04
19.05	Phytol, acetate	$C_{22}H_{42}O_2$	338	0.24
19.46	Dibutyl phthalate	$C_{16}H_{22}O_4$	278	0.16
19.93	Betulin	$C_{30}H_{50}O_2$	442	0.03
21.48	Ethyl iso-allocholate	$C_{26}H_{44}O_5$	436	0.01
22.62	Cyclodecasiloxane, eicosamethyl-	$C_{20}H_{60}O_{10}Si_{10}$	740	0.03
